# Supplementary material for: Horse immunization with short-chain consensus α-neurotoxin generates antibodies against broad spectrum of elapid venomous species
Source: Nat Commun. 2019 Aug 13;10:3642. doi: 10.1038/s41467-019-11639-2 (PMC6692343; doi:10.1038/s41467-019-11639-2)
Supplement: Supplementary file 2 — Reporting Summary [file 41467_2019_11639_MOESM2_ESM.pdf]

nature research

Corresponding author(s): Guillermo de la Rosa and Gerardo Corzo

Reporting Summary

Nature Research wishes to improve the reproducibility of the work that we publish. This form provides structure for consistency and transparency in reporting. For further information on Nature Research policies, see Authors & Referees and the Editorial Policy Checklist.

Statistical parameters

When statistical analyses are reported, confirm that the following items are present in the relevant location (e.g. figure legend, table legend, main text, or Methods section).

n/a	Confirmed	
☐	☒	The exact sample size (*n*) for each experimental group/condition, given as a discrete number and unit of measurement
☐	☒	An indication of whether measurements were taken from distinct samples or whether the same sample was measured repeatedly
☐	☒	The statistical test(s) used AND whether they are one- or two-sided<br>*Only common tests should be described solely by name; describe more complex techniques in the Methods section.*
☒	☐	A description of all covariates tested
☒	☐	A description of any assumptions or corrections, such as tests of normality and adjustment for multiple comparisons
☐	☒	A full description of the statistics including central tendency (e.g. means) or other basic estimates (e.g. regression coefficient) AND variation (e.g. standard deviation) or associated estimates of uncertainty (e.g. confidence intervals)
☒	☐	For null hypothesis testing, the test statistic (e.g. *F*, *t*, *r*) with confidence intervals, effect sizes, degrees of freedom and *P* value noted<br>*Give P values as exact values whenever suitable.*
☒	☐	For Bayesian analysis, information on the choice of priors and Markov chain Monte Carlo settings
☒	☐	For hierarchical and complex designs, identification of the appropriate level for tests and full reporting of outcomes
☒	☐	Estimates of effect sizes (e.g. Cohen's *d*, Pearson's *r*), indicating how they were calculated
☐	☒	Clearly defined error bars<br>*State explicitly what error bars represent (e.g. SD, SE, CI)*

Our web collection on statistics for biologists may be useful.

Software and code

Policy information about availability of computer code

Data collection	Prism (GraphPad, Inc., San Diego, CA) was used to calculate the data by non-linear regression. Detailed methodology is mentioned in relevant section in methods.
Data analysis	Prism (GraphPad, Inc., San Diego, CA) was used to calculate the data by non-linear regression. Detailed methodology is mentioned in relevant section in methods.

For manuscripts utilizing custom algorithms or software that are central to the research but not yet described in published literature, software must be made available to editors/reviewers upon request. We strongly encourage code deposition in a community repository (e.g. GitHub). See the Nature Research guidelines for submitting code & software for further information.

Data

Policy information about availability of data

All manuscripts must include a data availability statement. This statement should provide the following information, where applicable:

- Accession codes, unique identifiers, or web links for publicly available datasets
- A list of figures that have associated raw data
- A description of any restrictions on data availability

Provide your data availability statement here.

Field-specific reporting

Please select the best fit for your research. If you are not sure, read the appropriate sections before making your selection.

☒ Life sciences ☐ Behavioural & social sciences ☐ Ecological, evolutionary & environmental sciences

For a reference copy of the document with all sections, see nature.com/authors/policies/ReportingSummary-flat.pdf

Life sciences study design

All studies must disclose on these points even when the disclosure is negative.

Sample size	Five mice per group were used to determine the LD50 and ED50. We followed standards recommended by WHO published in the Progress in the characterization of venoms and standardization of antivenoms and the Guidelines for the production, control and regulation of snake antivenom immunoglobulins. Detailed methodology is mentioned in relevant section in methods.
Data exclusions	No data were excluded from the analyses.
Replication	LD50 and ED50 are determined by injecting five mice per group. Each group is injected intravenously with varying amount or volumes of toxin/venom or antivenoms, respectively, according to guidelines for the Guidelines published by WHO involved in the production, control and regulation of venoms and antivenoms.
Randomization	Only mice (either male or females) weighting 18-20 g were used in our LD50 and ED50 determination. Healthy castrated male horses (400 -500 kg) were used for antivenom production. Therefore, any further randomization protocol was required
Blinding	Only healthy mice and horses were considered in our analysis. Therefore, no blinding was performed.

Reporting for specific materials, systems and methods

Materials & experimental systems

n/a	Involved in the study
☐	☒ Unique biological materials
☐	☒ Antibodies
☒	☐ Eukaryotic cell lines
☒	☐ Palaeontology
☐	☒ Animals and other organisms
☒	☐ Human research participants

Methods

n/a	Involved in the study
☒	☐ ChIP-seq
☒	☐ Flow cytometry
☒	☐ MRI-based neuroimaging

Unique biological materials

Policy information about availability of materials

Obtaining unique materials	Venoms used in this study were from different sources. Certified, Naja melanoleuca (715.030 and 307.150), Naja katiensis (code not provided), Naja oxiana (911.040), Naja pallida (316.000), Naja nivea (524.010), Naja nubiae (101.030), Naja mossambica (505.010), Naja nigricollis (105.030), Naja haje (822.090) Naja kaouthia (506.000), Naja atra (920.100), Dendroaspis polylepis (218.020), Dendroaspis angusticeps (305.00), Dendroaspis viridis (815.050), Walterinnesia aegyptia (729.030) and Ophiophagus hannah (923.090) venoms were from Latoxan (Valence, France); Naja nivea (NNC019) was from SA Venom Suppliers (Louis Trichardt, South Africa); Naja naja naja (4NN9001) from Ventoxin; the australian venoms Oxyuranus scutellatus (OSS0319EAS, Cooktown, Australia), Pseudechis australis (PA0319EAS, Coen Cape York, Australia), Pseudechis colleti (PC0319EAS, Vergemont, Australia) were acquired through “La Nauyaca” (UMA INE/CITES/DFYFS-HERP-E-0003-MOR/98, Morelos, Mexico); venoms from Micrurus fulvius (08.31.10) and Micrurus tener tener (02.16.09) were from National Natural Toxins Research Center; and venoms from Micrurus laticollaris (two individuals), Micrurus browni, Micrurus diastema (two individuals), Micrurus distans, Micrurus nigrocinctus (two individuals), Micrurus surinamensis and sea snake Hydrophis (Pelamis) platura24 were obtained manually by milking multiple adult snakes at the “Instituto de Biotecnología” (UNAM, Mexico, permit SGPA/DGVS/010526/18) and “Instituto Clodomiro Picado” (UCR, Costa Rica, permit CICUA-021-17) serpentariums.

Antibodies

Antibodies used	100 microliters/well of Peroxidase-conjugated rabbit anti-horse immunoglobulins (Rockland, 208-4304).
Validation	Information of the antibody validation is available through manufacturer's online database (https://rockland-inc.com/store/IgG-Fraction-Antibodies-208-4304-O4L_1457.aspx). No further validation was done on the antibody in the reported experiments.

Animals and other organisms

Policy information about studies involving animals; ARRIVE guidelines recommended for reporting animal research

Laboratory animals	Mice of strain CD-1 (18-20 g) were purchased from Harlam, Mexico. Five to six years old castrated horses (400-500 kg) were purchased and maintained at Rancho Ojo de Agua, Puebla, México.
Wild animals	No wild animals were used.
Field-collected samples	All animals received regularly veterinary supervision and were maintained under good conditions and controlled environments. They received water and food ad libitum. Proper animal handling, in order to minimize distress and discomfort, was always conducted towards maximizing the animals welfare during experimentation. Animal handling was according to the Animal Care and Bioethics Committee at the "Instituto de Biotecnología" and "Instituto Clodomiro Picado", which supervised and approved all animal experiments
